# Deep Belief Network for Spectral–Spatial Classification of Hyperspectral Remote Sensor Data

**DOI:** 10.3390/s19010204

**Published:** 2019-01-08

**Authors:** Chenming Li, Yongchang Wang, Xiaoke Zhang, Hongmin Gao, Yao Yang, Jiawei Wang

**Affiliations:** 1 College of Computer and Information, Hohai University, Nanjing 211100, China; lcm@hhu.edu.cn (C.L.); wangyongchang@hhu.edu.cn (Y.W.); rcyyang@hhu.edu.cn (Y.Y.); 142006020223@hhu.edu.cn (J.W.); 2 School of Public Administration, Hohai University, Nanjing 211100, China; xkzhang@hhu.edu.cn

**Keywords:** hyperspectral image, deep learning, feature extraction, classification, remote sensors, multi-sensor fusion

## Abstract

With the development of high-resolution optical sensors, the classification of ground objects combined with multivariate optical sensors is a hot topic at present. Deep learning methods, such as convolutional neural networks, are applied to feature extraction and classification. In this work, a novel deep belief network (DBN) hyperspectral image classification method based on multivariate optical sensors and stacked by restricted Boltzmann machines is proposed. We introduced the DBN framework to classify spatial hyperspectral sensor data on the basis of DBN. Then, the improved method (combination of spectral and spatial information) was verified. After unsupervised pretraining and supervised fine-tuning, the DBN model could successfully learn features. Additionally, we added a logistic regression layer that could classify the hyperspectral images. Moreover, the proposed training method, which fuses spectral and spatial information, was tested over the Indian Pines and Pavia University datasets. The advantages of this method over traditional methods are as follows: (1) the network has deep structure and the ability of feature extraction is stronger than traditional classifiers; (2) experimental results indicate that our method outperforms traditional classification and other deep learning approaches.

## 1. Introduction

With the development of high-resolution optical sensors, hyperspectral remote sensing images are achieved, which consist of hundreds of different spectral bands of the same remote sensing scene. Hyperspectral remote images are essential tools for tasks such as target detection and classification because of these images’ advantage in describing ground truth information. Their applications vary and include agriculture, military, geology, and environmental science. Different land covers have various spectral curves due to the complexity of the composition of the earth’s surface. Hyperspectral images are rich in spectral information. Each pixel can produce a high-resolution curve. Traditional multispectral remote images use only a few bands to represent a complete spectral curve. However, dealing with hundreds of bands is also challenging [1]. Due to the large amount of hyperspectral remote sensing image data, the classification speed is slow. At the same time, the high spectral dimension of hyperspectral remote sensing images leads to the appearance of Hughes phenomenon.

Hyperspectral remote sensing images are collected by high-resolution optical sensors; the datasets used in this experiment were obtained by an airborne visible/infrared imaging spectrometer (AVIRIS) sensor and reflective optics system imaging spectrometer (ROSIS) sensor, respectively.

Classifying hyperspectral images is a common technique in discovering information in hyperspectral sensor data. Prior to image classification, dimension reduction is necessary because hyperspectral images contain a large amount of information. Neural networks and support vector machines (SVMs) [2] are extensively used in hyperspectral classification because of their potential in handling high-dimension data. They can manage most of the classification but cannot provide enriched information. This problem renders these algorithms limited in several areas.

In recent years, research on deep learning received considerable attention due to breakthroughs in many fields. The use of deep learning in classifying hyperspectral images can result in high accuracy [3]. There are several common models in deep learning, such as the deep neural network model and recurrent neural network model. They have their own representative networks, convolution neural network and recursive neural network, respectively, referred to as CNN and RNN. CNN effectively reduces the problem of a large number of parameters. The convolution core is used as the intermediary. After image convolution, the original position relationship still exists, while the parameters of the hidden layer of the image input layer are reduced by geometric multiples. The basic operation units of CNN are convolution, pooling, full connection, and recognition. The recursive neural network can also be called a forward neural network. Sample processing time is independent. In RNN, the output of neurons can act on themselves at the next time. The recursive neural network can be regarded as a neural network that transmits in time. To improve the accuracy of classification, this work proposes the application of a deep belief network (DBN) model to hyperspectral images. DBN is an algorithm proposed by Hilton in 2006 [4]. It is based on a neural network and developed using the hierarchical learning method, which learns input data layer by layer in an unsupervised manner. Each layer is created using a restricted Boltzmann machine (RBM). The features learned are regarded as the input of the subsequent layer. Finally, a softmax classifier used in the last layer fine-tunes the parameters of the network in a supervised manner and labels each pixel and the result of the classification. In other words, DBNs are obtained by stacking RBMs on one another so that the input to one layer is given by the hidden units of the adjacent layer, as if they were data, and adding a last discriminative layer [5].

A Boltzmann machine is powerful in unsupervised learning and can locate information hidden among data. Thus, it is suitable for data mining. A Boltzmann machine is a fully connected network. This structure extends the training time, thereby restricting the application of the network. An RBM and its learning algorithm can address the problems of deep neural networks, such as classification, regression, image feature extraction, and collaborative filtering.

Many variants of the RBM were developed since the machine was created. The convolutional RBM created by Reference [6] can extract large-scale features and exhibit good performance. An effective method using the TLCNN-RBM (convolutional neural network mixed restricted Boltzmann machine based on transfer learning) model for a small sample of voiceprint recognition was provided by Reference [7]. In Reference [8], a Gaussian RBM was proposed to learn multiple layers of features from small images, whereas, in Reference [9], a conditional RBM learned to represent spatial transformations using factored high-order Boltzmann machines.

The main contribution of this work is the development of the stacks of an RBM, hereafter called the DBN model. We modified the standard RBM and its learning algorithm. The processes can be regarded as pretraining and fine-tuning, wherein data are trained in mini-batches to optimize the loss function of the validation dataset. The framework learns deep features that model different ground-truth classes in hyperspectral images. We experimentally demonstrate that this generative feature learning for a spatial classifier (SC) or joint spectral–SC (JSSC) becomes effective, using the learned features to exhibit state-of-the-art performance on hyperspectral image classification.

The rest of this paper is organized as follows: in Section 2, the main ideas and the structure of the DBN are discussed in detail, and the proposed hyperspectral sensor data classification method is presented in combination with spectral information and the spatial context. In Section 3, experimental results are elaborated. Finally, the study is summarized in Section 4.

## 2. Methods

This section introduces the composition of RBM and the common DBN model, and then introduces the DBN classification model based on spatial information and joint spatial–spectral information.

### 2.1. RBM

An RBM is a random generative neural network composed of two layers. One layer comprises binary visible units, and the other comprises binary hidden units. An energy function was introduced to identify the state of the RBM, which was developed from the energy function of the Hopfield network in a nonlinear dynamic system. Therefore, the objective function of the system was transformed into an extreme value problem, and the RBM model could be easily analyzed [10].

An RBM is regarded as an Ising model; hence, its energy function is expressed as

(1) $$E(v,h;\theta )=-\sum_{ij}W_{ij}v_{i}h_{j}-\sum_{i}b_{i}v_{i}-\sum_{j}a_{j}h_{j},$$

where *θ* = (w,a,b) is the parameter of the RBM; $w_{ij}$ represents the value of the connection between the visible units v and the hidden units h; and $b_{i}$ and $a_{j}$ are bias terms of the visible and hidden units, respectively.

The conditional distributions of the hidden units *h* and the visible units *v* are expressed as

(2) $$P(h_{j}=1|v)=\frac{1}{1+\exp(-\sum_{i}W_{ij}v_{i}-a_{j})},$$

(3) $$P(v_{i}=1|h)=\frac{1}{1+\exp(-\sum_{j}W_{ij}h_{j}-b_{i})}.$$

The target function of the RBM focuses on the solution of the distribution of *h* and *v* and renders them as equal as possible. Thus, we calculated the K–L (Kullback-Leibler) distance of their distribution and then reduced it.

In determining the expectation of the joint probability, obtaining the normalization factor Z(*θ*) is difficult, and the time complexity will be $O(2^{m+n})$. Hence, Gibbs sampling was introduced to approximately reconstructed data. The learning of weights is expressed as

(4) $$\Delta w_{ij}=E_{data}(v_{i}h_{j})-E_{\mathrm{model}}(v_{i}h_{j}).$$

The subtracted value was equal to the expectation of the energy function of the input data and could be obtained. This value was equal to the expectation of the energy function of the model, which was obtained from Gibbs sampling.

Training the RBM through Gibbs sampling is time consuming. We commonly use the contrastive divergence (CD-k) algorithm, where k is equal to 1; thus, the training time of the RBM network improves [10].

The average sum of the gradients was approximated using the samples obtained from the conditional distributions, and Gibbs sampling was performed only once.

### 2.2. DBN

A DBN is a probability generation model that is opposite the traditional discriminative model. This network is a deep learning model that is stacked by RBM and trained in a greedy manner. The output of the previous layer was used as the input of the subsequent layer. Finally, a DBN network was formed.

DBN hierarchical learning was inspired by the structure of the human brain. Each layer of the deep network can be regarded as a logistic regression (LR) model.

The joint distribution function of x and $h^{k}$ in Layer l is

(5) $$p(x,h^{1},h^{2},\ldots ,h^{l})=(\prod_{k=0}^{l-2}P(h^{k}|h^{k+1}))P(h^{l-1},h^{l}).$$

The input data of the DBN model comprise the two-dimensional (2D) vector obtained during preprocessing. The RBM layers were trained one by one in pretraining. The succeeding visible variable was the duplicate of the hidden variable in the previous layer. The parameters transferred in layer-wise manner, and the features were learned from the previous layer. The LR in the highest layer was trained by fine-tuning, where the cost function was revised via back propagation to optimize the weights *w* [11].

**Figure 1:**
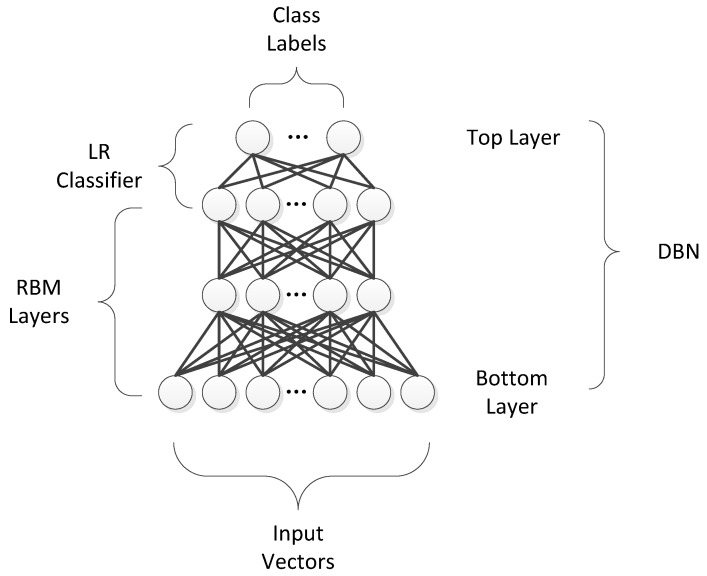
Architecture of a deep belief network (DBN).

The DBN architecture is shown in Figure 1.

Two steps are involved in the process of training a DBN model. Each RBM layer is unsupervisedly trained, the input should be mapped into different feature spaces, and information should be kept as much as possible. Subsequently, the LR layer is added on top of the DBN as a supervised classifier [12].

### 2.3. Proposed Method

Features are the raw materials for training and influence the performance of the final model. Theoretically, the more hidden layers are present, the more features the deep neural network (DNN) can extract, and the more complex the function learned. Accordingly, the DNN model can be described in detail. However, the DNN is gradually replaced by a shallow learning model, such as SVM and boosting, due to problems that occur when the weights *w* are initialized with a random number in a multi-layer network.

If the weights are set to be too large, then the training process will result in the local optimum. When the weights are set too small, gradient dispersion will occur, and the weights change gradually due to the small gradient. Obtaining the optimal solution is also difficult.

To address these problems, a layer-by-layer initialization of the deep neural network can obtain initial weights that are close to the optimal solution [13]. Layer-by-layer initialization is obtained through unsupervised learning, which can be conducted automatically.

On the basis of the DBN model, this work proposes a new method of classifying hyperspectral images. The basic DBN classification model involves preprocessing of data, pretraining, and fine-tuning. The difference between DBN and a neural network is the introduction of pretraining. The initial weight value can be close to the global optimization in the DBN model. Therefore, the greedy layer-wise supervised learning has better accuracy than does a neural network. In the supervised fine-tuning procedure, the mini-batch DBN model validates the learned features and the loss function to update the weights *w*. Parameters are trained in mini-batches every training epoch.

**Figure 2:**
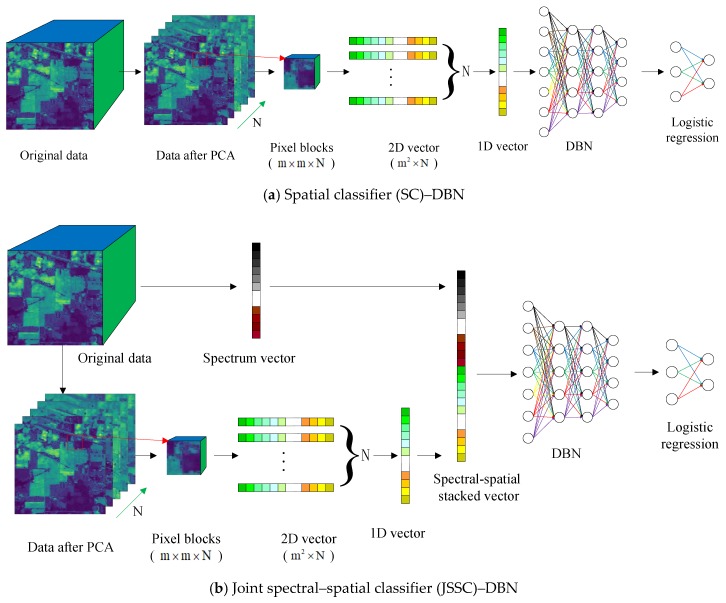
Process of hyperspectral classification based on DBN.

Hyperspectral sensor data comprise a spectral image cube that combines spectral and spatial information. Therefore, we compared the effect of two DBN structures, namely SC–DBN and JSSC–DBN. For SC–DBN, input data were the spatial information. Meanwhile, for JSSC–DBN, the input data comprised a new vector that combined the spectral and spatial information [14]. The entire process is presented in Figure 2.

#### 2.3.1. Spatial Classification

Spatial information plays an important role in classification when we attempt to improve accuracy. Before the spatial data are obtained, dimension reduction must be conducted on the hyperspectral remote images. Unlike spectral classification, which extracts spectral information from each pixel, SC cuts an image by window size, as shown in Figure 2a. Firstly, principal component analysis (PCA) dimension reduction was carried out to extract *n* main components of spectral information. Then, the neighborhood pixel blocks of m×m were extracted with the labeled pixels as the center, so that each pixel block contained spatial structure information, and the size of the pixel block was m×m×N. After that, the block of pixels was transformed into a 2D feature vector with the size of $m^{2}\times N$. Finally, the 2D feature vector was stretched to a one-dimensional (1D) vector with the size of $m^{2}N\times 1$.

Then, the 1D vector with spatial information was fed into the DBN model. We constructed each RBM layer that shared weighs with each sigmoidal layer. In each layer, CD-k was used in pretraining, so that weights could be updated by errors between the predicted classification results and the true land cover labels. Finally, spatial feature was learned layer by layer. Stochastic gradient descent was used to update the cost function in finetuning. In every epoch, the SC–DBN model calculated the cost on the validation set to make the loss as close as possible to the best validation loss. At the end, we tested it on the test set to obtain accuracy and kappa coefficient.

#### 2.3.2. Joint Spectral–Spatial Classification

Joint spectral and spatial classification (JSSC) was long applied in hyperspectral sensor data classification [15,16]. In our proposed work, a spectral–spatial classifier was introduced in the deep learning method. To fully use the spectral–spatial information, joint spectral–spatial classification places the spectral and spatial features together in a DBN classifier. Generally speaking, the pixels in the same spatial neighborhood have the same or similar spectral characteristics as the central pixels. Therefore, the neighborhood pixel blocks of m×m are extracted with the labeled pixels as the center, and each pixel block contains spatial structure information. Then, the pixel blocks are transformed into 2D feature vectors, and the 2D feature vectors are transformed into 1D vectors. Meanwhile, the 1D vector containing spectral information is extracted, whose length is equal to the number of spectral bands. The 1D vector with spatial information and the 1D vector containing spectral information are stitched together into a vector, which is the input of JSSC–DBN.

The spatial information of hyperspectral images has the correlation that the same object will occupy a certain space. Thus, the size of the window should be selected properly. We selected m×m data and placed an m×m×N neighbor region to feed a $m^{2}\times N$ vector into the input layer. We merged spatial data and spectral data into one input vector. Later on, regularization was used in principal component analysis (PCA) when we trained the parameters of the JSSC–DBN model. PCA was conducted to reduce the dimension of the hyperspectral image, while regularization could overcome overfit problem in training. The difference between JSSC and SC is that the former concatenates spatial and spectral vectors to form a new vector, which is then passed to the input layer.

The processing of the multi-layer RBM network in JSSC is discussed above. For the target dataset, the model analysis determined the accuracy of each class and the classification map at the end.

## 3. Experiments and Analysis

### 3.1. Dataset and Set-Up

**Figure 3:**
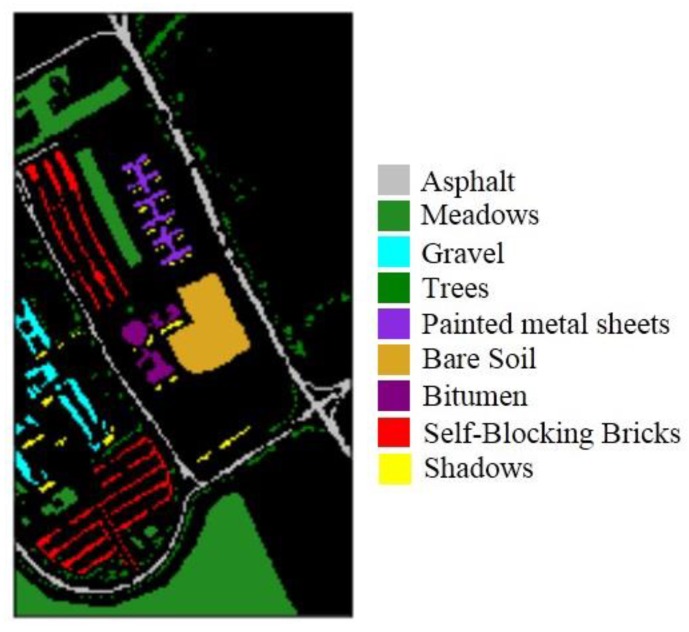
Pavia University representing nine classes.

We used common remote images to verify the effectiveness of the proposed method. One image came from the Pavia University dataset, which was captured by the ROSIS sensor. The ROSIS sensor is a compact, programmable imaging spectrometer based on a CCD (charge-coupied device) matrix detector array. The instrument is specially designed for monitoring water color and natural chlorophyll fluorescence, with the purpose of quantitatively extracting the distribution of pigments, suspended substances, and yellow substances in the marine environment [17]. The scene is sized 610 × 340 pixels and has 103 bands (after noisy bands are removed). The geometric resolution is 1.3 m with nine classes, namely asphalt, bitumen, gravel, sheet, bricks, shadows, meadows, soil, and trees, as shown in Figure 3. The total number of samples, training sets, validation sets, and test sets for each class is detailed in Table 1.

**Figure 4:**
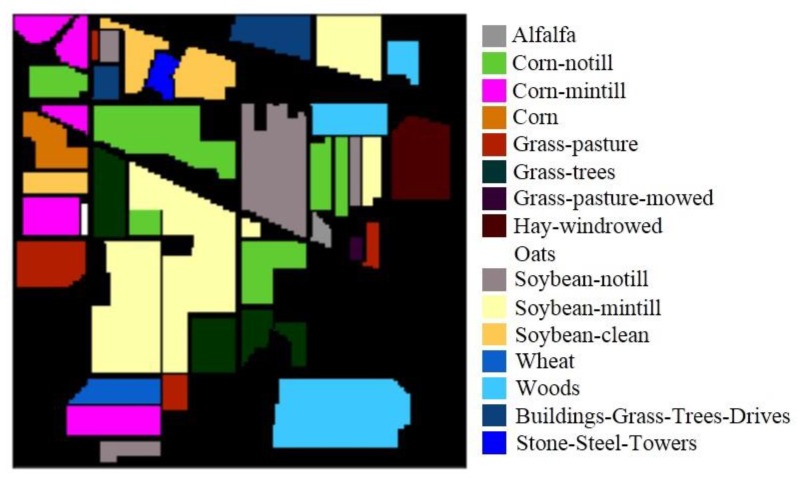
Indian Pines representing 16 classes.

Another example was the Indian Pines dataset, which is sized of 145 × 145 pixels and has 224 bands. The image was captured by the AVIRIS sensor. The AVIRIS sensor was flown for the first time in 1986 (first airborne images), and captured first science data in 1987; its data can provide a spatial resolution of 20 m and 224 spectral bands, covering a spectral range of 0.2–2.4 phenotypes, with a spectral resolution of 10 nm [18]. After de-noising of the original images, the remaining 200 bands were kept without water absorption for the experiments. Figure 4 shows the ground-truth objects. The total number of samples, training sets, validation sets, and test sets for the 16 ground-truth objects of Indian Pines are shown in Table 2.

In our experiment, we trained the proposed methods to investigate the influence of the parameters in the DBN network and to improve accuracy. To compare the results of SC and JSSC on the two datasets, we investigated the overall accuracy (OA), average accuracy (AA), and kappa coefficient. The program was run by Python libraries.

The preprocessing step included conducting PCA on the entire data and transforming the spectral and spatial information into a 2D vector. This step rendered the three-dimensional (3D) matrix into the vector, which could be the input of the DBN model.

To avoid potential autocorrelation issues, a validation dataset was added to the training and test data. The ratio of the training, validation, and testing was 6:2:2, as shown in Table 1 and Table 2. The original data were in matrix form but also needed to be normalized. The parameters of the proposed DBN network are shown in Table 3, and the window size of both datasets was 7 × 7. The parameters of the SVM were determined using the grid-search algorithm, and we set *c* to 10,000 and *g* to 10 [19].

We performed the experiments 100 times with the original randomized training samples to obtain the experimental results of SVM and the DBN classifier.

### 3.2. SC

**Figure 5:**
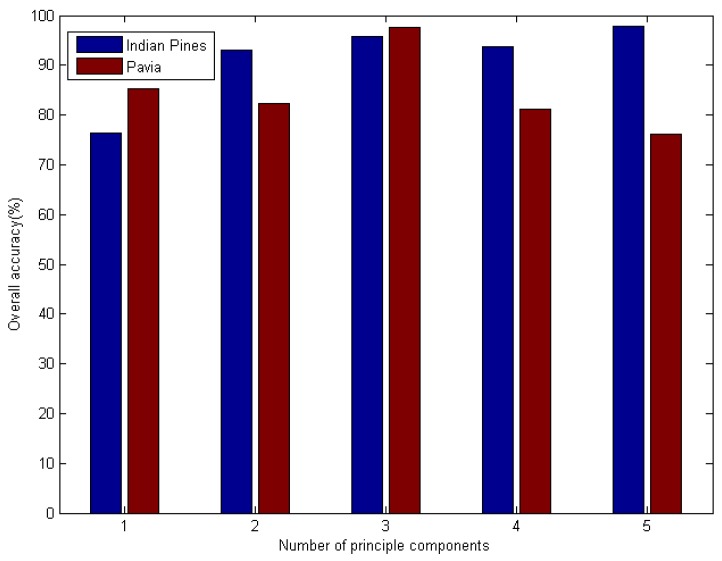
Effect of principal components (SC–DBN classifier).

In this part of the experiment, we began by classifying the hyperspectral sensor data using the SC–DBN method. We focused on the effect of the number of principals. The number of principal components was selected from 1 to 5. The pretraining epochs were set to 1000 for Indian Pines and 800 for Pavia University. The result is shown in Figure 5. Evidently, the optimal component was 5 for both datasets. Meanwhile, we investigated the influence of the number of hidden layers, which are also called “depths”. Similar experiments were performed on depths not exceeding 5.

The detailed classification accuracies and corresponding kappa coefficients of the DBN are shown in Table 4.

In SC–DBN, feature extraction is difficult. We selected the number of principals in range 5 and overall accuracy was considered as the measurement. According to Figure 5, the number of principal components influenced the accuracy of classification. For Indian Pines, The SC–DBN model performed best when *n* was 5. For Pavia University, the best number of principal components was 3.

**Figure 6:**
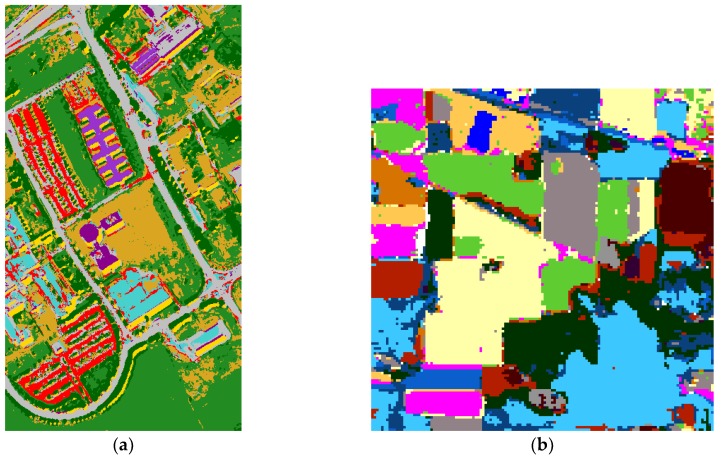
Spatial information-dominated classification result for Pavia University (**a**) and Indian Pines (**b**).

The entire image classification result of SC–DBN is shown in Figure 6. From the classification map, different classes are shown in several colors. For SC, the experiments could achieve accuracy of about 97.7% in Indian and 95.8% in Pavia. In summary, the classification results indicate that the DBN models performed well on the hyperspectral images. The edge of each class was not notably straight when compared with the ground truth.

### 3.3. JSSC

**Figure 7:**
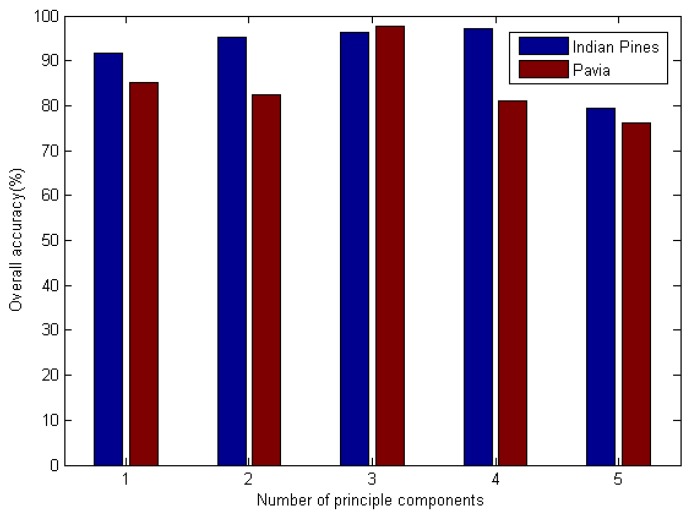
Effect of principal components (JSSC–DBN classifier).

In this section, we investigated the influence of the number of principal components and hidden layers on spectral–spatial information. Similarly, the number of principal components was researched for JSSC. The testing results of Indian Pines and Pavia datasets are shown in Figure 7. It shows the effect of the number of principal components when using the JSSC–DBN method to classify the hyperspectral sensor data. The best numbers of principal components for Indian Pines and Pavia University were 4 and 3, respectively.

For JSSC, the experiments could achieve overall accuracy of about 97.7% in Indian and 95.8% in Pavia. Therefore, the DBN model is a promising method for classifying hyperspectral images, whether using SC or JSSC.

**Figure 8:**
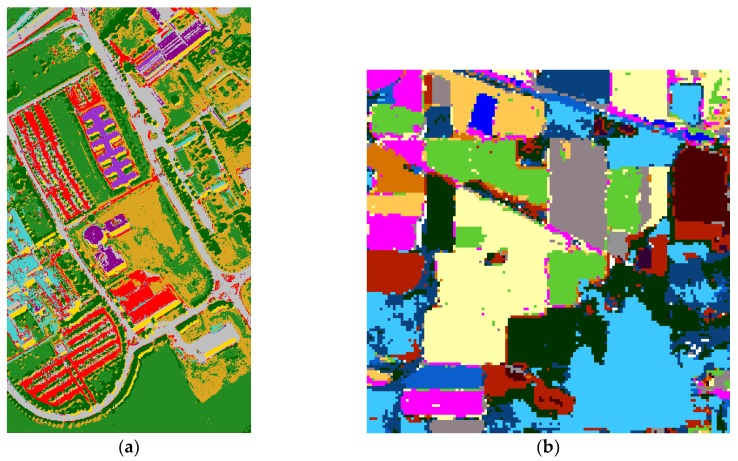
Joint-dominated classification result for Pavia University (**a**) and Indian Pines (**b**).

The joint-dominated classification maps on Pavia University and Indian Pines are shown in Figure 8.

In terms of the SVM-based method, spatial and spectral classification could also obtain high accuracy. The origin data were split into two parts for the verification of the classification results and calculation of the AA, thus comparing the SVM-based method and the proposed technique.

Detailed classification accuracies and corresponding kappa coefficients of each class are shown in Table 5. As the JSSC can extract more information than SC and SVM, it is unsurprising to find that JSSC performed the best among the three classifiers.

## 4. Conclusions and Discussion

In this work, we proposed a new hyperspectral image classification model based on DBN. The proposed model learns deep features during hyperspectral image classification. The framework of a DBN was introduced to classify spatial hyperspectral sensor image data on the basis of the DBN. Then, the improved method, which combines spectral and spatial information, was verified. After unsupervised pretraining and supervised fine-tuning, the DBN model could successfully learn the features. We added an LR layer on top for classifying the hyperspectral images. In comparison with the SVM-based method, the DBN model performed better in terms of accuracy and kappa coefficient. In addition, JSSC–DBN was proven to be the best classifier.

Therefore, the deep learning method improves the accuracy of hyperspectral classification. According to our results, we suggest that the DBN model be designed with 3–5 hidden layers, with each having no more than 100 hidden units.

In our future work, we will improve the DBN model regarding its accuracy and time consumption. Given the irreplaceable role of spectral–spatial feature extraction in the DBN model, further investigation should be devoted to parameter optimization of the deep learning framework. The DBN model runs slowly; thus, the PCA algorithm was selected to reduce the dimension of hyperspectral data, before being input into the DBN model designed in this paper for classification. However, the performance of the PCA algorithm in classification tasks is not ideal. In the future, we will continue improving the model in combination with the latest achievements in the field of dimensionality reduction algorithms.

## Figures and Tables

**Table 1:** Land cover classes and numbers in Pavia University.

#	Class	Samples	Training	Validation	Test
1	Asphalt	6631	3979	1326	1326
2	Meadows	18,649	11,189	3730	3730
3	Gravel	2099	1259	420	420
4	Trees	3064	1838	613	613
5	Painted metal sheets	1345	807	269	269
6	Bare Soil	5029	3017	1006	1006
7	Bitumen	1330	798	266	266
8	Self-blocking bricks	3682	2210	736	736
9	Shadows	947	569	189	189
**Total**		**42,776**	**25,666**	**8555**	**8555**

**Table 2:** Land cover classes and numbers in Indian Pines.

#	Class	Samples	Training	Validation	Test
1	Alfalfa	46	28	9	9
2	Corn-notill	1428	856	286	286
3	Corn-mintill	830	498	166	166
4	Corn	237	143	47	47
5	Grass-pasture	483	289	97	97
6	Grass-trees	730	438	146	146
7	Grass-pasture-mowed	28	16	6	6
8	Hay-windrowed	478	286	96	96
9	Oats	20	12	4	4
10	Soybean-notill	972	584	194	194
11	Soybean-mintill	2455	1473	491	491
12	Soybean-clean	593	355	119	119
13	Wheat	205	123	41	41
14	Woods	1265	759	253	253
15	Buildings-grass-trees-drives	386	232	77	77
16	Stone-steel-towers	93	55	19	19
**Total**		**10,249**	**6147**	**2051**	**2051**

**Table 3:** The deep belief network (DBN) network parameters.

Dataset	Number of Hidden Layers	Number of Hidden Layer Nodes	Pretrain Learning Rates	Fine-Tune Learning Rates
Indian Pines	3	310 × 100 × 100	0.01	0.001
Pavia	3	280 × 100 × 100	0.05	0.003

**Table 4:** Overall accuracy (OA), average accuracy (AA), and kappa coefficients of Indian Pines and Pavia University. SC—spatial classifier; JSSC—joint spectral–spatial classifier; SVM—support vector machine.

Dataset	Measurements	SC–DBN *n* = 3	JSSC–DBN *n* = 3	SVM
Indian Pines	OA (%)	95.81	96.29	85.71
	AA (%)	94.50	95.18	82.93
	Kappa (%)	95.22	95.78	83.26
Pavia	OA (%)	95.83	97.67	85.45
	AA (%)	94.67	96.79	80.33
	Kappa (%)	94.54	96.95	80.94

**Table 5:** Classification result on Indian Pines.

Class	SC–DBN (*n* = 4)	JSSC–DBN (*n* = 4)	SVM
Alfalfa	100	100	33.33
Corn-notill	92.71	96.87	94.44
Corn-mintill	86.93	94.01	77.84
Corn	95.56	100	88.89
Grass-pasture	97.03	100	91.09
Grass-trees	96.53	98.26	91.33
Grass-pasture-mowed	85.71	100	28.57
Hay-windrowed	100	100	97.94
Oats	100	100	33.33
Soybean-notill	86.01	98.93	84.46
Soybean-mintill	97.59	97.73	100
Soybean-clean	85.59	92.03	66.95
Wheat	95.83	97.91	91.67
Woods	99.21	98.41	91.67
Buildings-grass-trees-drives	80.25	89.23	27.16
Stone-steel-towers	100	100	0.00
Kappa (%)	92.82	96.88	83.26
Overall accuracy (%)	93.71	97.26	85.71
Average accuracy (%)	93.68	96.28	83.08
